# Controlling Tungiasis in an Impoverished Community: An Intervention Study

**DOI:** 10.1371/journal.pntd.0000324

**Published:** 2008-10-22

**Authors:** Daniel Pilger, Stefan Schwalfenberg, Jörg Heukelbach, Lars Witt, Norbert Mencke, Adak Khakban, Hermann Feldmeier

**Affiliations:** 1 Institute for Microbiology and Hygiene, Charité–University of Medicine, Campus Benjamin Franklin, Berlin, Germany; 2 Department of Community Health, School of Medicine, Federal University of Ceará, Fortaleza, Brazil; 3 Mandacaru Foundation, Fortaleza, Brazil; 4 Bayer HealthCare AG, Animal Health Division, Leverkusen, Germany; Oswaldo Cruz Foundation, Brazil

## Abstract

**Background:**

In Brazil, tungiasis is endemic in some resource-poor communities where various domestic and sylvatic animals act as reservoirs for this zoonosis. To determine the effect of control measures on the prevalence and intensity of infestation of human and animal tungiasis, a repeated cross-sectional survey with intervention was carried out.

**Methodology/Principal Findings:**

In a traditional fishing community in Northeast Brazil, humans and reservoir animals were treated, and premise-spraying using an insecticide was done, while a second fishing community served as a control. Both communities were followed up 10 times during a 12-month period. At baseline, prevalence of tungiasis was 43% (95% confidence interval [CI]: 35%–51%) and 37% (95% CI: 31%–43%) in control and intervention villages, respectively. During the study, prevalence of tungiasis dropped to 10% (95% CI: 8%–13%; p<0.001) in the intervention village, while the prevalence remained at a high level in the control village. However, after one year, at the end of the study, in both communities the prevalence of the infestation had reached pre-intervention levels. Whereas the intensity of infestation was significantly reduced in the intervention community (p<0.001), and remained low at the end of the study (p<0.001), it did not change in the control village.

**Conclusion/Significance:**

Our study shows that a reduction of prevalence and intensity of infestation is possible, but in impoverished communities a long-lasting reduction of disease occurrence can only be achieved by the regular treatment of infested humans, the elimination of animal reservoirs, and, likely, through environmental changes.

**Trial Registration:**

Controlled-Trials.com ISRCTN27670575

## Author Summary

Tungiasis is a disease caused by the sand flea *Tunga penetrans*, a parasite prevalent in many impoverished communities in developing countries. The female sand flea penetrates into the skin of animals and humans where it grows rapidly in size, feeds on the host's blood, produces eggs which are expelled into the environment, and eventually dies in situ. The lesions become frequently superinfected and the infestation is associated with considerable morbidity. Clearly, tungiasis is a neglected disease of neglected populations. We investigated the impact of a package of intervention measures targeted against on-host and off-host stages of *T. penetrans* in a fishing community in Northeast Brazil. These measures decreased disease occurrence only temporarily, but had a sustained effect on the intensity of the infestation. Since infestation intensity and morbidity are correlated, presumably the intervention also lowered tungiasis-associated morbidity. Control measures similar to the ones used in this study may help to effectively control tungiasis in impoverished communities.

## Introduction

Tungiasis is a parasitic disease caused by the sand flea *Tunga penetrans*. Female fleas penetrate into the epidermis where they undergo a process of so-called neosomy and expel several hundred eggs into the environment. After a period of six weeks, the parasite dies in situ and is sloughed off the epidermis by tissue repair mechanisms [1].

During the last decades growing urbanization and improved housing has resulted in a reduction of prevalence. Today, the occurrence of tungiasis is confined to resource-poor communities located at the coast or in the rural hinterland, and to slums of rapidly growing urban agglomerations in Latin America, the Caribbean and Sub-Saharan Africa [2],[3]. In these settings prevalences range between 15% and 51% [2], [4]–[6]. Since prevalence, intensity of infestation, and morbidity are positively related [7], debilitating and disfiguring sequels are common in resource-poor rural and urban communities. Tungiasis is clearly a neglected disease of marginalized populations [8],[9].

Although by its nature a self-limiting disease, tungiasis causes considerable morbidity [10],[11]. Fissures, ulcers, gangrene, lymphedema, deformation and loss of nails and auto-amputation of digits are known sequels [10]. In non-immune individuals tungiasis is a risk factor for tetanus [12],[13]. Superinfection of the lesions is virtually constant [14],[15], and a variety of aerobic and anaerobic bacteria have been isolated from embedded sand fleas [16],[17].

Beside humans, *T. penetrans* parasitizes a range of domestic animals, such as dogs, cats, pigs and rodents [18],[19]. In Brazil, dogs and cats act as important reservoirs for the intra- and peridomiciliary transmission of sand fleas [18],[20]. When humans live in close contact with infested animals, the risk of infestation is high and the intensity of infestation is high [20].

The control of tungiasis in a resource-poor population with interventions targeted at the human and the animal population has never been described. Here we present the results of an intervention performed in collaboration with public health services in an endemic community in northeast Brazil. The results show that combining treatment of humans with treatment of animals and focal spaying of an insecticide reduced prevalence and intensity of infestation.

## Methods

### Study area and population

To evaluate the effect of multiple interventions on the prevalence of tungiasis, two endemic communities were selected in Ceará State, northeast Brazil (Balbino and Pedro de Souza). Both communities are situated in Cascavél Municipality about 60 km south of Fortaleza, the state capital. The fishing communities are separated about 6 km and located within sand dunes near the Atlantic Ocean, showing little fluctuation of their population. The two communities are very similar with regard to demographic, crowding, social and economic characteristics. Houses are located on rather large compounds surrounded by fences and built on sandy soil. The quality of housing is poor and streets are not paved. Kitchens are indoors or consist of open stalls on the compound. Both communities are integrated in the national Family Health Program (“Programa da Saúde da Família”) and served by community health care workers (“agentes comunitários de saúde”). During the study, the closest primary health care center was located in another community, some 10 km away.

In October 2002, Balbino was inhabited by 148 families with a total population of 630. The community of Pedro de Souza comprised 251 individuals in 60 families. Inhabitants of all age groups were eligible for the study, provided they had spent at least four days per week in the village during the last three months. Dogs and cats were included if they were born in the villages or had lived there for at least two months. Pigs, goats, sheep, cows and horses, other species of domestic animals occurring in the villages were previously excluded to be animal reservoirs of *T. penetrans* in this setting [20].

### Study design

The study was conceived as a repeated cross-sectional survey with intervention. To assess the impact of interventions on the prevalence and intensity of infestation, two complete villages were compared. In the community Balbino various interventions were implemented while the Pedro de Souza community served as a control. Interventions were coordinated and implemented by the Mandacaru Foundation (Fortaleza, Brazil) in collaboration with the Health Secretariat of Cascavel Municipality. Between November 2002 and November 2003, a total of 10 surveys were planned and carried out in each community. The villages were visited during identical periods according to pre-defined dates with a maximum delay of 10 days between intervention and control community.

The study started in November 2002, in the middle of the dry season when the prevalence of tungiasis peaks [21]. During the preparatory phase, contact was made with community leaders, and the objectives of the study were explained. Censuses of the human and the animal population were performed and all houses mapped using a global positioning system (GPS). All data were collected by door-to-door surveys. The primary outcome was the prevalence of tungiasis (dichotomous), and the secondary outcome intensity of infestation (continuous).

All surveys were carried out by the same investigators (S.S., L.W.), accompanied by community health agents. During the surveys the human and the animal population were carefully examined for the presence of embedded sand fleas, according to previously established guidelines. In humans the entire body was examined (except the genitals) to identify any ectopic lesions [22]. In dogs and cats examination focused on paws, abdomen and muzzle, the topographic areas most commonly affected [18]. The following findings were considered to be diagnostic for human as well as animal tungiasis [1]: a red-brownish spot with a diameter of 1–3 mm with visible posterior segments of the penetrated flea (early stage); a circular whitish lesions with a diameter of 4–10 mm with a central black dot (mature stage), round black crust surrounded by necrotic tissue (late stage with dead parasite). Typical residuals in the epidermis, lesions altered through manipulation (such as partially or totally removed fleas leaving a characteristic crater-like sore in the skin), and suppurative lesions (caused by the use of nonsterile instruments), were recorded as well. Lesions were differentiated into viable, dead and manipulated lesions. At each assessment, participants were also asked about any adverse events that may have occurred in consequence of the intervention.

Clinical experience shows that infestation with more than ten sand fleas oftentimes result in considerable morbidity. We therefore considered infestation with up to five embedded fleas as low, between six and as 10 moderate and >10 lesions as a high intensity of infestation, in analogy to a previously used classification [23]. In animals the stratification was <10, 11–20 and >20, respectively.

After baseline examination in November 2002, the control measures were carried out in the intervention village (Balbino). From November 2002 through January 2003, from all infested individuals embedded sand fleas were extracted every two to three weeks by experienced health care professionals under sterile conditions. The remaining sore was treated with an antibiotic ointment. During the same period all cats and dogs were treated with trichlorphone 97% in oily solution (Neguvon, Bayer do Brasil, São Paulo, Brazil) or neck collars impregnated with propoxur and flumethrin (Kiltix, Bayer Bayer do Brasil, São Paulo, Brazil). In case of loss of neck collars these were substituted at the next survey. In February 2003, deltamethrin was used for focal premise treatment. Focal spraying was performed by trained personnel of the Health Secretariat of Cascavel Municipality. The insecticide was sprayed on the ground next to the houses targeting areas in which off-host development of *T. penetrans* was suspected to occur, such as preferred whereabouts of dogs and cats, and shady places under trees [24], or inside houses in the case of a sandy floor. Focal premise treatment using insecticides was repeated twice during a period of six weeks. Table 1 summarizes the type and the period of interventions.

**Table 1:** Study design: type, onset and duration of intervention against *T. penetrans*.

Intervention	Period	Type of intervention	Targeted against
1	November 02–end of January 03	Surgical extraction of sand fleas^a^	Imbedded adult sand fleas
2	November 02–end of January 03	On-host treatment of animals^b^	Invading adult flea, imbedded adults
3	Mid January 03–March 03	Focused premise treatment using insecticides^c^	Off-host stages

a every two to three weeks.

b On-host treatment with Kiltix and Neguvon.

c Repeated after 30 days.

### Ethical considerations

The study protocol was approved by the Ethical Review Board of the Federal University of Ceará Fortaleza, Brazil (Protocol no. 195/02). In addition, an ad hoc ethical committee, consisting of physicians, community members and professionals of the Health Secretariat of Cascavel Municipality approved the study protocol. Informed written consent was obtained from all study participants. In the case of minors, written consent was obtained from the minors and their carers. Written consent was also obtained from pet owners. At the end of the study surgical treatment of tungiasis was offered to affected individuals in both communities.

### Statistical analysis

Data were entered in Epi Info version 6.04d (CDC, Atlanta, USA), checked for entry errors and transferred to SPSS 11.04 for Macintosh (SPSS Inc., Chicago, IL, USA) for analysis. The χ^2^ test was employed to determine the significance of difference of proportions between population groups, and between the intervention and control communities. For the comparison of point prevalences within one community, the McNemar test was used. Infestation intensities were compared by the Mann-Whitney test. The prevalence ratio [PR] for tungiasis and the significance of differences in the relative distribution of tungiasis were calculated in contingency tables. To calculate the relative prevalence reduction Balbino and Pedro de Souza were regarded as one study population, and belonging to either community was considered an exposure variable. The relative prevalence reduction was calculated as follows: prevalence of tungiasis in Pedro de Souza–prevalence of tungiasis in Balbino/prevalence of tungiasis in Pedro de Souza.

## Results

The intervention community was considerable bigger, but socio-demographic characteristics were similar in both communities. Table 2 summarizes baseline characteristics of the study populations.

**Table 2:** Demographic characteristics of the two study populations.

Human population	Balbino Intervention village	Pedro de Souza Control village
Age (median, range)	27 (1–87)	27 (1–82)
Sex ratio (female/male)	1.25	1.07
Total population	630	281
Number of individuals meeting inclusion criteria at baseline	597	234
Percentage of individuals examined during the nine follow-up examinations (median, interquartile range)	77% (64–84%)	60% (52–62%)
Number of animals meeting inclusion criteria at baseline*	123–197	34–36
Percentage of animals examined during the nine follow-up examinations (median, interquartile range)	63% (45–79)	87% (74–100)

*: dogs and cats were infested; data of both species combined.

**Figure 1:**
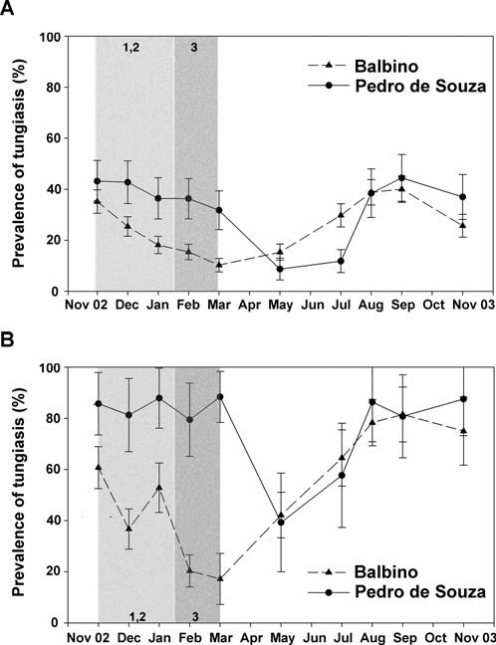
Point prevalences of tungiasis during study period. Explanatory text: Grey areas mark the intervention period in (A) the human population and (B) the animal population. Numbers indicate type and onset of interventions (see Table 1).

At baseline (November 2002) the prevalence of tungiasis in the human population was slightly higher in Pedro de Souza, the control village (43%, 95% confidence interval [CI]: 35–51%), than in Balbino, the intervention village (37%, 95% CI: 31–43%;). However, the difference was not significant (Figure 1A; p = 0.11).

At the first follow-up in December 2002 –after completion of the first cycle of intervention measures 1 and 2 (see Table 1)– the prevalence of tungiasis dropped to 25% (95% CI: 35–51%) in Balbino, while it remained unchanged in Pedro de Souza (pre-intervention versus one month post-intervention p = 0.05 and p = 0.47, respectively; Figure 1A). In the intervention village prevalence continued to decrease to 18% (95% CI: 15–22%) in January 2003 (p = 0.001), 15% (95% CI: 12–18%) in February (p = 0.17), and 10% (95% CI: 8–13%) in March (p = 0.001; all p compared to the preceding survey). During the same period no significant reduction in prevalence of tungiasis was noted in Pedro de Souza (Figure 1A). Here, prevalence in March 2003 remained as high as 36% (95% CI: 29–44%). During the rainy season a strong reduction in prevalence was observed in the control community, too. Prevalence decreased significantly from 36% (95% CI: 29–44%) in March to 9% (95% CI: 4–13%) in May (p<0.001).

With the beginning of the dry season (June–July 2003) prevalence started to rise in both communities, and prevalence curves were almost parallel. By November 2003 one year after beginning of the study, prevalences in both communities had reached the pre-intervention level.

During the 12-month study period, the prevalence curves of animal tungiasis showed a similar pattern as compared to human tungiasis, with higher baseline values in Pedro de Souza (86%, 95% CI: 71–100%) than in Balbino (64%, 95% CI: 52–75%; *p* = 0.02) and an impressive decrease in prevalence during the rainy season (Figure 1B). However, the variation of measurements was considerably higher than in the human population.

The PR of tungiasis showed no significant association at the beginning of the intervention in November 2002 (PR = 0.81, 95% CI: 0.65–1.03; *p* = 0.11). After cessation of the intervention (March 2003) the PR had decreased significantly in Balbino, as compared to Pedro de Souza (PR = 0.28, 95% CI: 0.2–0.39; *p*<0.001). At the end of the study in November 2003 the PR remained slightly but significantly lower in the intervention village (PR = 0.69, 95% CI: 0.52–0.93; *p* = 0.017). The prevalence reduction in Balbino was 55% and 19% in March and November 2003, respectively.

**Figure 2:**
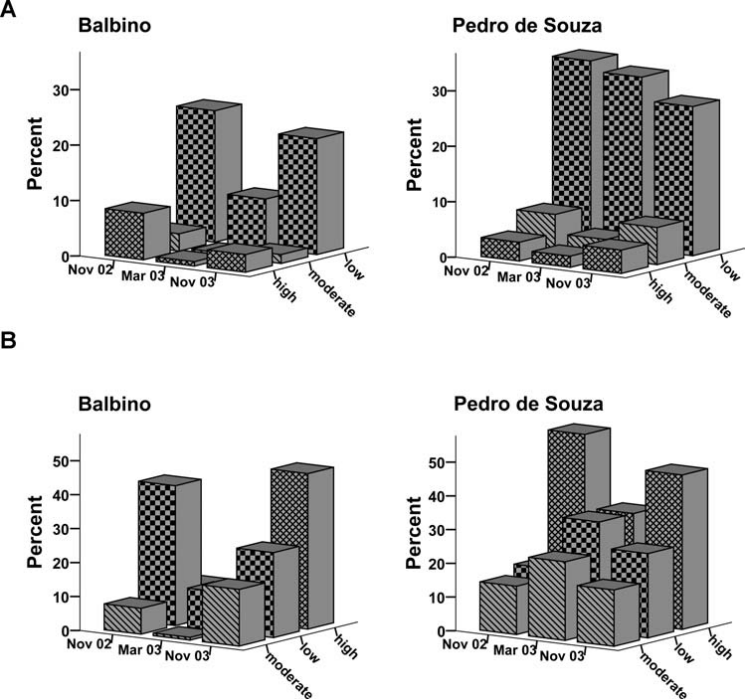
Intensity of infestation during study period. Explanatory text: Intensity of infestation in humans (A) and animals (B); pre-intervention, immediately after the intervention, and after one year of follow-up.

In the intervention population, the prevalence of individuals with a high and a moderate intensity of infestation decreased significantly from pre-intervention (November 2002) to four months after intervention (March 2003): prevalence of individuals with high intensity 8.4% (95% CI: 6–11%) versus 0.8% (95% CI: 0.02–1.5%; p<0.001) and with moderate intensity 3.3% (95% CI: 1.6–5%) versus 0.6% (95% CI: 0.001–1.2%), p = 0.002 (Figure 2A). In contrast, no significant reduction was observed in the control village: 3% (95% CI: 0.4–6%) versus 2% (95% CI: 0–4%; p = 0.99) and 6.8% (95% CI: 2–11%) versus 3.4% (95% CI: 0.4–6%), respectively (p = 0.25; Figure 2A).

At the end of the study (November 2003) the prevalence of heavy and moderate infested individuals remained significantly lower in Balbino as compared to the pre-intervention level: 8.4% (95% CI: 6–11%) versus 3% (95% CI: 1–5%) after intervention (p = 0.001), and 3.3% (95% CI: 1.6–5%) versus 1.6% (95% CI: 0.3–2.8%; p = 0.013), respectively. In contrast, at the end of the study in Pedro de Souza the proportion of heavily and moderate infested individuals was even higher than at baseline: 3% (95% CI: 0.4–6%) versus 4% (95% CI: 0.5–7%; p = 0.25) and 6.8% (95% CI: 2–11%) versus 6.7% (95% CI: 2–11; p = 0.99), respectively (Figure 2A). Similar, the total number of embedded sand fleas per person was significantly reduced in the intervention village, from a median of 18 in November 2002 to a median number of one in March 2003 (p<0.001) and remained significantly lower in November 2003 (median of five lesions; p<0.001 compared to baseline).

There were no significant differences in the reduction of intensity of infestation in the animal population (Figure 2B).

No adverse events related to the intervention were recorded. However, as our therapy did not differ from the treatment done by most community members themselves, we assume that people did not report any minor adverse events associated with surgical extraction (such as pain and superficial bleeding).

## Discussion

The high prevalence of tungiasis in endemic areas and the important morbidity associated with this parasitic skin disease call for the implementation of control measures. As a first step to prove that successful intervention is possible, we determined the impact of repeated rounds of surgical extraction of embedded sand fleas in humans in combination with on-host treatment of dogs and cats, and focal spraying of an insecticide on the premises. Our study shows that the interventions were effective to control tungiasis in the short-term but failed to show an impact on the long run. This is reflected by a minuscule reduction of the prevalence ratio at the end of the study, i.e. 12 months after start of interventions.

Several factors seem to be responsible for the re-increase of prevalence to baseline level in the intervention village at the end of the study. Firstly, the backbone of the intervention, the surgical extraction of embedded sand fleas, obviously has several shortcomings. As inhabitants of endemic areas rarely remove embedded sand fleas in a systematic manner–it is time-consuming, painful and often results in superinfection [25]– individuals could have abstained from the treatment. As a consequence, the human reservoir of *T. penetrans* may not have diminished as intended [23].

Presumably the inflammatory reaction of the skin at sites of embedded sand fleas facilitates penetration [25]. Since the barrier function of the epidermis is not immediately reconstituted after sand fleas have been taken out (the sore produced by the surgical manipulation even might temporarily increase the surface of the skin particular susceptible to penetration), the idea that i) the reduction of the parasite burden would prevent re-infestation, and that ii) by consequence, the number of eggs expelled into the environment would reduce transmission, might be an invalid assumption. Actually, people at risk for immediate re-infestation could be more susceptible to the infestation with *T. penetrans* after extraction and may only profit from the surgical extraction after complete healing of the skin.

We suggest that the prevention of infestation, rather than the surgical extraction of already embedded sand fleas, may interrupt transmission more effectively. Zanzarin, a plant-based repellent, has been shown to effectively prevent the infestation with *T. penetrans* in areas with high attack rates [26]. This compound would be an ideal candidate for prophylaxis but was not available in Brazil when the study was designed.

Secondly, many cats and dogs remained infested despite the on-host treatment (Figure 1B). By consequence these animals continued spreading *T. penetrans* and contributed to ongoing transmission in the community [27]. After on-host intervention had been stopped, more and more animals became re-infested, with an even higher prevalence at the end of the study as compared to baseline data (Figure 1B). Assumably, these animals carried sand fleas to the compounds of their owners where they fuelled peri- and intradomiciliary transmission [20]. Actually, in both communities the strongest increase in prevalence of tungiasis in humans followed a strong increase in prevalence in animals (Figures 1 A, B). Recently developed on-host products, such as a combination of imidacloprid and permethrin (Advantix), effectively prevented infestation with *T. penetrans* in animals and lowered parasite burden [27]. Again, this product was not available when the study was conceived.

Finally, focused premise treatment with deltamethrin aimed to interrupt the off-host cycle of *T. penetrans* [18],[28]. For an optimal efficacy, focal spraying has to be applied at all sites where off-host development takes part in the soil, which means, that those sites have to be identified first. Due to delivery problems and constraints in qualified personnel, focal premise treatment only started in January, i.e., very late in the seasonal cycle of *T. penetrans*. In addition, there was no expertise to examine soil samples for developmental stages of sand fleas. Obviously, spraying of breeding sites is better done before the parasite population has expanded, i.e., at the beginning of the dry season [21]. However, the significant reduction in prevalence after the implementation of this intervention seems to have booster the protective effect of the preceding interventions.

When transmission of *T. penetrans* is altered–e.g. through an intervention–the intensity of infestation reflects the infestation rates individuals have experienced during the last months [7]. Thus, intensity of infestation is a better outcome measure to evaluate the impact of an intervention than assessment of the prevalence. In addition, it is a better proxy of morbidity reduction, as morbidity is significantly correlated to intensity of infestation [7].

In contrast, prevalence merely measures absence or presence of infestation at a given point of time and will therefore rapidly increase if the attack rate is high, such as at the beginning of the dry season. Prevalence does not allow inferring on decreasing attack rates prior to the assessment since embedded sand fleas and their remains are visible for up to three months [1].

Our study shows that in the intervention community a prolonged effect on the intensity of infestation of the human population occurred. This means, that despite a persistent high prevalence of tungiasis, attack rates were reduced. However, this effect was not observed in the animal population (Figure 2B). This indicates that the intervention measures applied were not very effective in reducing the parasite burden in dogs and cats.

Any assessment of intervention methods is hampered by the characteristic seasonal variation of tungiasis. Disease occurrence decreases as soon as the rainy season starts and re-increases with the beginning of the dry season [21]. We therefore opted to include a control community where no intervention was done. Although both fishing communities were selected by their similarity with respect to demographic, physical and socio-cultural characteristics, one cannot exclude that they have differed in an unknown factor of epidemiological relevance, an additional, hitherto neglected animal reservoir. Such a factor could be responsible for different dynamics in prevalence between Balbino and Pedro de Souza during the year. However, different dynamics in the same epidemiological setting have never been reported and the similar development of prevalence towards the end of the study renders such an explanations unlikely.

One major problem in the interpretation of data arises from the differences in population size between the two communities. This difference bares the possibility that significances in the changes of prevalence and/or intensity of infestation in the control village during the study period were not detected due to the smaller sample size.

Non-participation may have biased the assessment of point-prevalences during the observation period. For surveys with a low participation, it is conceivable that over- or under-representation of infested individuals has skewed the results towards higher or lower prevalence. Fluctuation of participation was particularly high in animals, indicating that point prevalences in the animal population were likely to be biased.

Another shortcoming of the study is that the study design does not allow identifying the relative effective of the three interventions applied in Balbino village. To address such an issue, various communities would have to be included, in each of which a particular intervention has to be performed. For operational reasons and financial constraints this could not be done.

Due to the operational nature of the study, we did not include any multivariate analysis. The study was not designed as a typical trial and thus did not include detailed collection of data on possible confounders. We aimed to describe whether an intervention has any effect rather than identifying independent factors for success or failure.

Off-host stages of *T. penetrans* develop best in dry soil or in dusty soil containing organic material [9],[21],[29]. Measures aiming to interrupt the off-host development should therefore focus on physically changing the environment in which eggs, pupae, and larva develop. This can be done through paving streets, cementing floors, and eliminating uncontrolled disposal of waste in public areas and private compounds [30],[31]. However, theses interventions require substantial funds and are beyond the economic capabilities of most communities where tungiasis is endemic.

Although the present study was conducted in a rural fishing community, it is conceivable that interventions are also applicable to urban settings where the animal reservoirs are similar [18].

Our study is a first step in the exploration of possible control measures against *T. penetrans*. Further studies are needed to assess the full potential of putative interventions. To do so, a cluster-randomized phased implementation study with various communities phasing-in specific interventions would be an ideal approach [32].

The Brazilian Unified Health System (SUS) with a network of primary health care clinics, many located in endemic communities, would offer a convenient way to coordinate such a study. Randomized clinics could implement intervention measures, such as prevention of infestation by application of Zanzarin, surgical extraction of embedded fleas, distribution of animal treatment, and coordination of premise spraying with insecticides. This would allow identifying the most effect measure over a prolonged period of time and controlling for seasonal variation [32].

## Supporting Information

Ethics S1 Ethics Approval
(0.41 MB PDF)

Checklist S1 CONSORT Checklist
(0.06 MB DOC)

Protocol S1 Study Protocol
(0.24 MB DOC)

## References

[1] Eisele M, Heukelbach J, Van Marck E, Mehlhorn H, Meckes O (2003). Investigations on the biology, epidemiology, pathology and control of *Tunga penetrans* in Brazil: I. Natural history of tungiasis in man.. Parasitol Res.

[2] de Carvalho RW, de Almeida AB, Barbosa-Silva SC, Amorim M, Ribeiro PC (2003). The patterns of tungiasis in Araruama township, state of Rio de Janeiro, Brazil.. Mem Inst Oswaldo Cruz.

[3] Wilcke T, Heukelbach J, Sabóia Moura RC, Kerr-Pontes LR, Feldmeier H (2002). High prevalence of tungiasis in a poor neighbourhood in Fortaleza, Northeast Brazil.. Acta Trop.

[4] Chadee DD (1998). Tungiasis among five communities in south-western Trinidad, West Indies.. Ann Trop Med Parasitol.

[5] Muehlen M, Feldmeier H, Wilcke T, Winter B, Heukelbach J (2006). Identifying risk factors for tungiasis and heavy infestation in a resource-poor community in northeast Brazil.. Trans R Soc Trop Med Hyg.

[6] Ugbomoiko US, Ofoezie IE, Heukelbach J (2007). Tungiasis: high prevalence, parasite load, and morbidity in a rural community in Lagos State, Nigeria.. Int J Dermatol.

[7] Kehr JD, Heukelbach J, Mehlhorn H, Feldmeier H (2007). Morbidity assessment in sand flea disease (tungiasis).. Parasitol Res.

[8] Heukelbach J (2005). Tungiasis.. Rev Inst Med Trop São Paulo.

[9] Heukelbach J, Oliveira FA, Hesse G, Feldmeier H (2001). Tungiasis: a neglected health problem of poor communities.. Trop Med Int Health.

[10] Feldmeier H, Eisele M, Saboia-Moura RC, Heukelbach J (2003). Severe tungiasis in underprivileged communities: case series from Brazil.. Emerg Infect Dis.

[11] Feldmeier H, Kehr JD, Poggensee G, Heukelbach J (2006). High exposure to *Tunga penetrans* (Linnaeus, 1758) correlates with intensity of infestation.. Mem Inst Oswaldo Cruz.

[12] Litvoc J, Leite RM, Katz G (1991). Aspectos epidemiológicos do tétano no estado do São Paulo (Brasil).. Rev Inst Med Trop São Paulo.

[13] Tonge BL (1989). Tetanus from chigger flea sores.. J Trop Pediatr.

[14] Feldmeier H, Heukelbach J, Eisele M, Sousa AQ, Barbosa LM (2002). Bacterial superinfection in human tungiasis.. Trop Med Int Health.

[15] Obengui (1989). [Tungiasis and tetanus at the University Hospital Center in Brazzaville].. Dakar Med.

[16] Fischer P, Schmetz C, Bandi C, Bonow I, Mand S (2002). *Tunga penetrans*: molecular identification of *Wolbachia* endobacteria and their recognition by antibodies against proteins of endobacteria from filarial parasites.. Exp Parasitol.

[17] Heukelbach J, Bonow I, Witt L, Feldmeier H, Fischer P (2004). High infection rate of *Wolbachia* endobacteria in the sand flea *Tunga penetrans* from Brazil.. Acta Trop.

[18] Heukelbach J, Costa AM, Wilcke T, Mencke N, Feldmeier H (2004). The animal reservoir of *Tunga penetrans* in severely affected communities of North-East Brazil.. Med Vet Entomol.

[19] Ugbomoiko US, Ariza L, Heukelbach J (2008). Pigs are the most important animal reservoir of *Tunga penetrans* (jigger flea) in rural Nigeria.. Tropical Doctor (in press).

[20] Pilger D, Schwalfenberg S, Heukelbach J, Witt L, Mehlhorn H (2008). Investigations on the biology, epidemiology pathology and control of *Tunga penetrans* in Brazil VII. The importance of animal reservoirs for human infestation.. Parasitol Res.

[21] Heukelbach J, Wilcke T, Harms G, Feldmeier H (2005). Seasonal variation of tungiasis in an endemic community.. Am J Trop Med Hyg.

[22] Heukelbach J, Wilcke T, Eisele M, Feldmeier H (2002). Ectopic localization of tungiasis.. Am J Trop Med Hyg.

[23] Muehlen M, Heukelbach J, Wilcke T, Winter B, Mehlhorn H (2003). Investigations on the biology, epidemiology, pathology and control of *Tunga penetrans* in Brazil. II. Prevalence, parasite load and topographic distribution of lesions in the population of a traditional fishing village.. Parasitol Res.

[24] Witt L, Heukelbach J, Schwalfenberg S, Ribeiro RA, Harms G (2007). Infestation of Wistar rats with *Tunga penetrans* in different microenvironments.. Am J Trop Med Hyg.

[25] Feldmeier H, Eisele M, Van Marck E, Mehlhorn H, Ribeiro R (2004). Investigations on the biology, epidemiology, pathology and control of *Tunga penetrans* in Brazil: IV. Clinical and histopathology.. Parasitol Res.

[26] Feldmeier H, Kehr JD, Heukelbach J (2006). A plant-based repellent protects against *Tunga penetrans* infestation and sand flea disease.. Acta Trop.

[27] Klimpel S, Mehlhorn H, Heukelbach J, Feldmeier H, Mencke N (2005). Field trial of the efficacy of a combination of imidacloprid and permethrin against Tunga penetrans (sand flea, jigger flea) in dogs in Brazil.. Parasitol Res.

[28] Gordon RM (1941). The jigger flea.. Lancet.

[29] White GB, Cook G, Zumla A (2003). Ectoparasites.. *Manson's* Tropical Diseases.

[30] Heukelbach J, Oliveira FA, Feldmeier H (2003). [Ectoparasitoses and public health in Brazil: challenges for control].. Cad Saude Publica.

[31] Ugbomoiko US, Ariza L, Ofoezie IE, Heukelbach J (2007). Risk factors for tungiasis in Nigeria: Indentification of targets for effective intervention.. PLoS Negl Trop Dis.

[32] Moulton LH, Golub JE, Durovni B, Cavalcante SC, Pacheco AG (2007). Statistical design of THRio: a phased implementation clinic-randomized study of a tuberculosis preventive therapy intervention.. Clin Trials.

