# Supplementary material for: Controlling Tungiasis in an Impoverished Community: An Intervention Study
Source: PLoS Negl Trop Dis. 2008 Oct 22;2(10):e324. doi: 10.1371/journal.pntd.0000324 (PMC2565488; doi:10.1371/journal.pntd.0000324)
Supplement: Ethics S1 — Ethics Approval (0.41 MB PDF) [file pntd.0000324.s001.pdf]

Universidade Federal do Ceará
Comitê de Ética em Pesquisa

Of. Nº 276/02 Fortaleza, 25 de outubro de 2002
Protocolo nº 195/02
Pesquisador responsável: Dr. Jorg Heukebach
Deptº./Serviço: Fundação de Educação e Saúde Mandacaru
Título do Projeto: "Tunguíase (bicho de pé): controle em uma comunidade severamente afetada no Estado do Ceará"

Levamos ao conhecimento de V.Sª. que o Comitê de Ética em Pesquisa e do Complexo Hospitalar da Universidade Federal do Ceará – COMEPE, dentro das normas que regulamentam a pesquisa em seres humanos, do Conselho Nacional de Saúde – Ministério da Saúde, Resolução nº196 de 10 de outubro de 1996 e Resolução nº 251 de 07 de agosto de 1997, publicadas no Diário Oficial, em 16 de outubro de 1996 e 23 de setembro de 1997, respectivamente, aprovou o projeto supracitado na reunião do dia 24 de outubro de 2002.

Atenciosamente,

Dr. Fernando A. Frota Bezerra
Coordenador do Comitê
de Ética em Pesquisa
COMEPE/UFC
