# Supplementary material for: Controlling Tungiasis in an Impoverished Community: An Intervention Study
Source: PLoS Negl Trop Dis. 2008 Oct 22;2(10):e324. doi: 10.1371/journal.pntd.0000324 (PMC2565488; doi:10.1371/journal.pntd.0000324)
Supplement: Protocol S1 — Study Protocol (0.24 MB DOC) [file pntd.0000324.s003.doc]

| 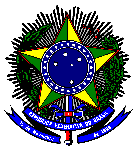 | MINISTÉRIO DA SAÚDE - Conselho Nacional de Saúde - Comissão Nacional de Ética em Pesquisa - CONEP  **FOLHA DE ROSTO PARA PESQUISA ENVOLVENDO SERES HUMANOS**  **( versão outubro/99 ) Para preencher o documento, use as indicações da página 2.** | | | | | | | | |
| --- | --- | --- | --- | --- | --- | --- | --- | --- | --- |
| **1.** Projeto de Pesquisa:  **Tunguíase (*bicho de pé*): controle em uma comunidade severamente afetada no Estado do Ceará** | | | | | | | | | |
| **2.** Área do Conhecimento (Ver relação no verso)  CIÊNCIAS DA SAÚDE | | | | | | **3.** Código:  4 | **4.** Nível: ( Só áreas do conhecimento 4 )  (E) Epidemiológico | | |
| **5.** Área(s) Temática(s) Especial (s) (Ver fluxograma no verso)  MEDICINA | | | | | | **6.** Código(s):  4 01 | **7.** Fase: (Só área temática 3) I ( ) II ( )  III ( ) IV ( ) | | |
| **8.** Unitermos: ( 3 opções )  TUNGUÍASE, EPIDEMIOLOGIA, CONTROLE | | | | | | | | | |
| SUJEITOS DA PESQUISA | | | | | | | | | |
| **9.** Número de sujeitos 620  No Centro : | | | | **10.** Grupos Especiais : <18 anos ( ) Portador de Deficiência Mental ( ) Embrião /Feto ( ) Relação de Dependência (Estudantes , Militares, Presidiários, etc ) ( ) Outros ( ) Não se aplica ( X ) | | | | | |
| PESQUISADOR RESPONSÁVEL | | | | | | | | | |
| **11.** Nome: JORG HEUKELBACH | | | | | |  | | | |
| **12.** Identidade:  7495 CRM-CE | | | **13.** CPF.: 646.710.543-53 | | | **19.**Endereço (Rua, n.º ):  RUA JOSÉ VILAR DE ANDRADE 257 | | |  |
| **14.** Nacionalidade:  ALEMÃ | | | **15.** Profissão:  MÉDICO | | | **20.** CEP:  60833-830 | **21.** Cidade:  FORTALEZA | | **22.** U.F.  CE |
| **16.** Maior Titulação:  DOUTORADO | | | **17.** Cargo  COORDENADOR DE PESQUISA | | | **23.** Fone:  (85) 2733031 | **24.** Fax  (85) 2733031 | | |
| **18.** Instituição a que pertence:  FUNDAÇÂO DE EDUCAÇÂO E SAÚDE MANDACARU | | | | | |  | **25.** Email:  [SAMSA@MCANET.COM.BR](mailto:SAMSA@MCANET.COM.BR) | | |
| **Termo de Compromisso:** Declaro que conheço e cumprirei os requisitos da Res. CNS 196/96 e suas complementares. Comprometo-me a utilizar os materiais e dados coletados exclusivamente para os fins previstos no protocolo e a publicar os resultados sejam eles favoráveis ou não. Aceito as responsabilidades pela condução científica do projeto acima.  Data: _______/_______/_______ ______________________________________  Assinatura | | | | | | | | | |
| INSTITUIÇÃO ONDE SERÁ REALIZADO | | | | | | | | | |
| **26.** Nome:  PREFEITURA DE CASCAVEL | | | | | | **29.** Endereço (Rua, nº): | | | |
| **27.** Unidade/Órgão:  SECRETARIA DE SAÚDE | | | | | | **30.** CEP: | **31.** Cidade:  CASCAVEL | | **32.** U.F.  CE |
| **28.** Participação Estrangeira: Sim ( ) Não ( X ) | | | | | | **33.** Fone (85) 334-1470; -2444 | **34.** Fax. (85) 334-1470 | | |
| 35. Projeto Multicêntrico: Sim ( ) Não ( X ) Nacional ( ) Internacional ( ) ( Anexar a lista de todos os Centros Participantes no Brasil ) | | | | | | | | | |
| **Termo de Compromisso ( do responsável pela instituição ) :**Declaro que conheço e cumprirei os requisitos da Res. CNS 196/96 e suas Complementares e como esta instituição tem condições para o desenvolvimento deste projeto, autorizo sua execução  Nome: JOSÉ POLICARPO BARBOSA ___________________________________________ Cargo: SECRETÁRIO DE SAÚDE_  Data: _______/_______/_______ ___________________________________  Assinatura | | | | | | | | | |
| **PATROCINADOR Não se aplica ( X )** | | | | | | | | | |
| **36.** Nome: | | | | | | **39.** Endereço | | | |
| **37.** Responsável: | | | | | | **40.** CEP: | **41.** Cidade: | | **42.** UF |
| **38.** Cargo/Função: | | | | | | **43.** Fone: | **44.** Fax: | | |
| COMITÊ DE ÉTICA EM PESQUISA - CEP | | | | | | | | | |
| **45.** Data de Entrada:  _____/_____/_____ | | | | | **46.** Registro no CEP: | **47.** Conclusão: Aprovado ( )  Data: ____/_____/_____ | **48.** Não Aprovado ( )  Data: _____/_____/_____ | | |
| **49.** Relatório(s) do Pesquisador responsável previsto(s) para:  Data: _____/_____/____ Data: _____/_____/_____ | | | | | | | | | |
| Encaminho a CONEP:  **50.** Os dados acima para registro ( ) **51.** O projeto para apreciação ( )  **52.** Data: _____/_____/_____ | | | | | | **53.** Coordenador/Nome  ________________________________  Assinatura | | **Anexar o parecer consubstanciado** | |
| COMISSÃO NACIONAL DE ÉTICA EM PESQUISA - CONEP | | | | | | | | | |
| **54.** Nº Expediente :  **55.** Processo : | | **56.**Data Recebimento : | | | | **57.** Registro na CONEP: | | | |
| **58. Observações:** | | | | | | | | | |

**FLUXOGRAMA PARA PESQUISAS ENVOLVENDO SERES HUMANOS (JAN/99)**

|  | **CEP Aprovação** |  |
| --- | --- | --- |

| GRUPO I **Código - Áreas Temáticas Especiais** | GRUPO II **Código - Área Temática Especial** | | GRUPO III **Todos os outros** que não se enquadrem  em áreas temáticas especiais |
| --- | --- | --- | --- |
| **I . 1.** Genética Humana  **I . 2.** Reprodução Humana  **I.. 4.** Novos Equip, insumos e dispositivos^(*)^  **I. 5.** Novos procedimentos  **I. 6.** Populações Indígenas  **I. 7.** Biossegurança  **I. 8.** Pesquisas com cooperação estrangeira  **I. 9.** A critério do CEP | **II. 3.** Novos Fármacos, Vacinas e  Testes Diagnósticos ^(*)^ | |  |
| (para apreciação)     (para banco de dados)  **Enviar:**  - Protocolo completo  - Folha de Rosto  - Parecer Consubstanciado | | **Enviar:**  - Folha de Rosto  - Parecer Consubstanciado (para acompanhamento) | **Enviar:**  Relatório Trimestral com  Folhas de Rosto |
|  | | CONEP |  |

**CÓDIGO – ÁREAS DO CONHECIMENTO ( Folha de Rosto Campos 2 e 3 )**

| 1. **CIÊNCIAS EXATAS E DA TERRA**    1. – MATEMÁTICA    2. – PROBABILIDADE E ESTATÍSTICA    3. - CIÊNCIA DA COMPUTAÇÃO    4. - ASTRONOMIA    5. - FÍSICA    6. - QUÍMICA    7. - GEOCIÊNCIAS    8. - OCEANOGRAFIA | **2 - CIÊNCIAS BIOLÓGICAS (*)**  2.01 - BIOLOGIA GERAL  2.02 - GENÉTICA  2.03 - BOTANICA  2.04 - ZOOLOGIA  2.05 - ECOLOGIA  2.06 - MORFOLOGIA  2.07 - FISIOLOGIA  2.08 - BIOQUÍMICA  2.09 - BIOFÍSICA  2.10 - FARMACOLOGIA  2.11 - IMUNOLOGIA  2.12 - MICROBIOLOGIA  2.13 - PARASITOLOGIA  2.14 - TOXICOLOGIA | **3 - ENGENHARIAS**  3.01 - ENGENHARIA CIVIL  3.02 - ENGENHARIA DE MINAS  3.03 - ENGENHARIA DE MATERIAIS E METALÚRGICA  3.04 - ENGENHARIA ELÉTRICA  3.05 - ENGENHARIA MECÂNICA  3.06 - ENGENHARIA QUÍMICA  3.07 - ENGENHARIA SANITÁRIA  3.08 - ENGENHARIA DE PRODUÇÃO  3.09 - ENGENHARIA NUCLEAR  3.10 - ENGENHARIA DE TRANSPORTES  3.11 - ENGENHARIA NAVAL E OCEÂNICA  3.12 - ENGENHARIA AEROESPACIAL |
| --- | --- | --- |
| **4 - CIÊNCIAS DA SAÚDE (*)**  4.01 – MEDICINA  4.02 – ODONTOLOGIA  4.03 – FARMÁCIA  4.04 – ENFERMAGEM  4.05 – NUTRIÇÃO  4.06 - SAÚDE COLETIVA  4.07 – FONOAUDIOLOGIA  4.08 – FISIOTERAPIA E TERAPIA OCUPACIONAL  4.09 – EDUCAÇÃO FÍSICA | **5 - CIÊNCIAS AGRÁRIAS**  5.01 - AGRONOMIA  5.02 - RECURSOS FLORESTAIS E ENGENHARIA FLORESTAL  5.03 - ENGENHARIA AGRÍCOLA  5.04 - ZOOTECNIA  5.05 - MEDICINA VETERINÁRIA  5.06 - RECURSOS PESQUEIROS E ENGENHARIA DE PESCA  5.07 - CIÊNCIA E TECNOLOGIA DE ALIMENTOS | **6 - CIÊNCIAS SOCIAIS APLICADAS**  6.01 - DIREITO  6.02 - ADMINISTRAÇÃO  6.03 - ECONOMIA  6.04 - ARQUITETURA E URBANISMO  6.05 - PLANEJAMENTO URBANO E REGIONAL  6.06 - DEMOGRAFIA  6.07 - CIÊNCIA DA INFORMAÇÃO  6.08 - MUSEOLOGIA  6.09 - COMUNICAÇÃO  6.10 - SERVIÇO SOCIAL  6.11 - ECONOMIA DOMÉSTICA  6.12 - DESENHO IDUSTRIAL  6.13 - TURISMO |
| **7 - CIÊNCIAS HUMANAS**  7.01 – FILOSOFIA  7.02 – SOCIOLOGIA  7.03 – ANTROPOLOGIA  7.04 – ARQUEOLOGIA  7.05 – HISTÓRIA  7.06 – GEOGRAFIA  7.07 – PSICOLOGIA  7.08 – EDUCAÇÃO  7.09 - CIÊNCIA POLÍTICA  7.10 – TEOLOGIA | **8 - LINGÜÍSTICA, LETRAS E ARTES**  8.01 - LINGÜÍSTICA  8.02 - LETRAS  8.03 - ARTES | **(*) NÍVEL** : **( Folha de Rosto Campo 4 )**  **(P)** **P**revenção  **(D) D**iagnóstico  **(T) T**erapêutico  **(E)** **E**pidemiológico  **(N)** Não se aplica |

**(*) OBS:** - As pesquisas das áreas temáticas 3 e 4 ( novos fárrmacos e novos equipamentos ) que dependem de licença de importação da **ANVS/MS**, devem obedecer ao seguinte fluxo- Os projetos da área 3 que se enquadrarem simultaneamente em outras áreas que dependam da aprovação da **CONEP**, e os da área 4 devem ser enviados à **CONEP**, e esta os enviará à **ANVS/MS** com seu parecer.

- Os projetos exclusivos da área 3 aprovados no CEP ( Res. CNS 251/97 – item V.2 ) deverão ser enviados à ANVS pelo patrocinador ou pesquisador.

Fundação de Educação e Saúde Mandacaru

Tunguíase ("*bicho de pé"*):

controle em uma comunidade severamente afetada no Estado do Ceará

*Projeto de pesquisa*

1. **Sinopse**

A tunguíase ("bicho de pé") é hiperendêmica em muitas comunidades pobres do nordeste brasileiro. Apesar disso, não existem medidas de intervenção estabelecidas.

# Esse projeto consiste em realizar medidas de intervenção para controlar a tunguíase em uma comunidade de pescadores. Essas medidas consistem na extração sistemática de pulgas penetradas na pele do ser humano e educação em saúde.

# Os resultados desse estudo fornecerão dados para a implantação de medidas de controle eficientes e efetivas aplicáveis também a outras comunidades pobres no Brasil. Além disso, colaborarão na integração dessas medidas no sistema de saúde existente.

1. **Objetivos**

- controlar a tunguíase em uma comunidade severamente afetada
- avaliar a efetividade e a eficiência de medidas de intervenção contra a tunguíase

**3. Introdução**

A tunguíase é uma doença ectoparasitária causada pela penetração da pulga fêmea *Tunga penetrans* na epiderme do seu hospedeiro e hipertrofia subseqüente.

Originalmente, a pulga é encontrada na América Latina e nos países do Caribe. O ectoparasita foi introduzido na África tropical em 1872 por um barco viajando do Brasil para Angola carregado de areia contaminada^1-4^. Hoje, a tunguíase é distribuída amplamente na América Latina, Caribe e África sub-sahariana^1-3,5-18,18-42^.

As pulgas adultas vivem livremente no meio ambiente, mas a fêmea penetra na epiderme do hospedeiro que pode ser o homem, cão, gato, porco, rato ou qualquer outro animal mamífero.^1,10,18,20,43-45^. Taxas de prevalência em cães, gatos e ratos em uma comunidade de pescadores no Estado do Ceará atingiram mais que 50% (Heukelbach et al., unpublished observation). Obviamente, animais domésticos infectados contribuem para altas taxas de ataque em seres humanos.

A infestação normalmente é limitada aos pés. As áreas de predileção são a região periungueal dos dedos, mas a penetração da pulga pode ocorrer em qualquer parte do corpo. Em crianças pequenas, lesões ectópicas foram observados nas mãos, cotovelos, pescoço, ânus e genitais ^46^.

Dentro de 24 horas após a penetração, a área começa a apresentar sinais de irritação e dois a três dias depois também a doer. Eritema e edema se desenvolvem ao redor da lesão. A pulga demora cerca de 8 a 12 dias para "amadurecer". Durante esse processo, o abdômen hipertrofia e atinge até um centímetro de diâmetro contendo 200 ovos^4,15,47^. Após eliminar os ovos, a pulga morre^47^. Depois, a lesão desseca *in situ* e os debris são expulsos.

Sem tratamento apropriado, infecção secundária com bactérias patogênicas é inevitável ^48^. Em áreas com cobertura vacinal baixa, o tétano é uma complicação comum em crianças^18,40^. Sepse, linfedemea, gangrena, perda de unha e auto-amputação de dígitos foram descritos^20,21,49-52^.

Em comunidades pobres do Brasil, Nigéria e Trinidad e Tobago, taxas de prevalência variaram entre 21% e 83% ^21,37,49,53^. Em todos os estudos, a prevalência em crianças era significantemente mais alta de que em adultos, com um pique na faixa etária de 5 a 10 anos. Em um levantamento em uma favela em Fortaleza, Estado do Ceará, infecção e patologia severa também foram mais comuns em crianças^54^. Se isso acontece devido à exposição menor dos indivíduos de maior faixa etária, à extração imediata após penetração ou à imunidade adquirida, nunca foi pesquisado. Consistentemente, a prevalência é mais alta em meninos do que meninas, provavelmente, porque os meninos estão mais expostos à *T. penetrans*^30,49^.

A terapia consiste na retirada da pulga com uma agulha estéril e aplicação tópica de um antibiótico. Se a pulga rompe na tentativa de retirada com uma agulha, por exemplo, poderá ocorrer inflamação severa.

Um relato de caso indica o tratamento sucessivo com dose única de ivermectina 0.2 mg/kg peso corporal^55^. De fato, o tratamento com ivermectina oral e tópica foi descrito como eficaz contra ectoparasitoses humanas como pediculose, escabiose e larva migrans cutânea^56-59^. Casos de tunguíase tratados com albendazol foram observados (J. Heukelbach, unpublished observation). Tiabendazol oral de 25 mg/kg de peso corporal durante 10 a 12 dias foi usado com eficácia em vários pacientes com tunguíase generalizada ^20,60^.

Banho de imersão de patas de cães com metrifonate 0.2% e injeção subcutânea de ivermectina (0.2 mg/kg peso corporal) foi relatado como efetivo na medicina veterinária ^10^. Outros autores relataram tratamento efetivo com pincelamento das patas de cães com neguvon 4%^61^.

A doença é conhecida há muito tempo, mas métodos de tratamento, prevenção e controle nunca foram avaliados de uma forma adequada.

4. Área de estudo

O estudo será realizado em Balbino, uma comunidade de pescadores no município de Cascavel, cerca de 50km ao sul de Fortaleza. Balbino é uma comunidade relativamente isolada, localizada perto da praia, com 620 habitantes. A população vive principalmente da pesca.

Em julho de 2001, um censo foi realizado, e a população inteira examinada para a presença de doenças parasitárias de pele. Foi encontrado que 61% da população apresentava pelo menos uma das seguintes ectoparasitoses: pediculose, escabiose, tunguíase ou larva migrans cutânea. Taxas de prevalência da tunguíase foram particularmente altas: mais de que 50% da população apresentou pulgas penetradas. As crianças são as mais acometidas: 76% na faixa etária de cinco a nove anos.

O contato com os líderes comunitários é bem estabelecido, e a comunidade já cooperou em vários estudos.

A área de estudo pode ser vista como modelo para as muitas outras comunidades pobres do nordeste brasileiro.

A área de estudo que serve como controle será a comunidade de Barra Velha no mesmo Município. Essa comunidade possui de cerca de 300 habitantes e tunguíase é também endêmica. Barra Velha tem características sócio-econômicas e geográficas similares à comunidade de Balbino.

**5. Desenho do estudo**

O estudo será composto por várias partes: atividades preparatórias, exame "baseline" antes da intevenção, a intervenção e o seu monitoramento, e a medida dos "outcomes" durante e após a finalização do estudo.

5.1 Atividades preparatórias:

As atividades preparatórias incluem a informação da população e a preparação do estudo na comunidade.

- - **sensibilização da população**Reuniões com a comunidade serão realizadas para informar a população sobre os objetivos do estudo. Será enfatizado que a participação é voluntária e que ninguém sofrerá de desvantagem por causa de não-participação. Informações sobre os objetivos serão distribuídas para todos os domicílios.
  - **consentimento pós-informação**Consentimento pós-informação será obtido de todos os participantes e donos de animais usando a ficha em anexo 1. O termo de consentimento será obtido pelos pesquisadores na oportunidade de visita das famílias em casa.
  - **recrutamento de auxiliares locais**Auxiliares locais serão recrutados para assistência no registro, exame e tratamento das pessoas e animais.

5.2 Exame "baseline"

Antes de começar a intervenção, o exame "baseline" será realizado colhendo as seguintes informações:

- **Censo de todos os habitantes e casas**Todas as famílias, indivíduos e casas serão registrados e possíveis fatores de risco documentados (como moradia, status socioeconômico, processo saúde-doença etc.). A comunidade inteira será mapeada usando um sistema móvel de GPS.
- **Prevalência da tunguíase**A prevalência da tunguíase na população humana será investigada. Isso inclui a documentação da intensidade de infecção (número médio de lesões por paciente, distribuição de estágios clínicos, razão de lesões vitais/não vitais e de lesões manipuladas/não manipuladas), e distribuição geográfica de famílias de alto risco.

# A diagnóstico da tunguíase será feito clinicamente. O corpo inteiro será examinado, excluindo as áreas genitais. Os seguintes achados são considerados patognomônicos para a tunguíase em animais e humanos: um ponto vermelho-amarronzado de um a três mm de diâmetro com segmentos posteriores da pulga penetrada visíveis (estágio inicial), lesões circulares brancas de quatro a dez mm de diâmetro com um ponto preto central apresentando os segmentos posteriores da pulga (estágio maduro), "casca preta" (a pulga morta circundade de tecido necrotizado, estágio final), como também lesões manipuladas pelo paciente ou dono do animal (pulgas parcialmente ou completamente removidas deixando uma lesão caracterísitca na pele como também lesões super-infectadas purulentas causadas pelo manipulação com instrumentos não estéreis). As lesões serão classificadas utilizando-se um sistema recentemente desenvolvido (*Fortaleza Classification*).

- ***Comunidade de controle***A comunidade de Barra Velha, no mesmo município, será incluída no estudo como controle. Essa comunidade apresenta caracterísiticas epidemiológicas e sócio-econômicas similares à comunidade de Balbino.Todos os habitantes dessa comunidade serão examinados mensalmente durante o período de um ano.
  Após esse período, o tratamento em massa será realizado com ivermectina e albendazol para diminuir a taxa de infestação por pediculose, escabiose e verminoses intestinais.
- ***Conhecimento sobre a tunguíase e o processo saúde-doença***O conhecimento sobre a tunguíase e o processo saúde-doença será investigado utilizando-se um questionário padronizado, de múltipla escolha e pré-testado. Todos os chefes de família serão interrogados. Na comunidade de controle, o mesmo questionário será aplicado.
- ***Pulgas em flea traps***Flea traps (armadilhas para pulgas) serão colocados em áreas de infestação alta. Após uma semana, serão examinados pela presença de *T. penetrans*, e o número de pulgas capturadas será documentado.

***5.3 Intervenção/tratamento***

Imediatamente após o baseline, os próximos passos serão realizados:

- ***Tratamento dos indivíduos***Para erradicar o reservatório humano, todos os indivíduos com lesões ativas de tunguíase (i.e. parasita vivo) serão tratados. O tratamento consiste da desinfecção da área com álcool, aplicação de lidocaína spray para anestesia local, retirada de pulgas penetradas com uma agulha estéril e aplicação de pomada antibiótica. Essa intervenção será repetida a cada três dias durante um período de três semanas. Posteriormente o tratamento será realizado uma vez por semana durante mais três semanas. A retirada das pulgas será realizada em cooperação com pessoas experientes da comunidade.
- ***Educação em saúde***Educação em saúde será realizada e será repassada informação sobre a biologia, patologia e tratamento da doença, além da informação de como retirar pulgas penetradas e encorajamento para inspeção regular dos pés.

***5.4 Monitoramento durante o "follow-up"***

O período de follow-up durará 12 meses. Os seguintes dados serão colhidos:

- ***Prevalência***Todos os habitantes de Balbino serão examinados mensalmente até 12 meses após o baseline.
- ***Taxas de prevalência na comunidade controle***Todos os habitantes da comunidade controle serão examinados mensalmente durante o período de um ano.

***5.5 Medidas de "outcome"***

Os seguintes dados serão usados para descrever a eficiência da intervenção:

- ***Tunguíase na população humana***Taxas de prevalência e severidade de doença na população antes, durante e após a intervenção serão comparados. Fatores de risco também serão investigados e comparados aos dados antes da intervenção.
  Conhecimento sobre a infecção e o processo saúde-doença serão comparados antes e após 12 meses de educação em saúde.
- ***Comunidade de controle***Para excluir que a variação sazonal da tunguíase influencia os outcome measures em Balbino, a variação das taxas longitudinais de prevalência serão comparadas à variação dessas taxas na comunidade de controle, Barra Velha.
  Para medir o impacto da educação em saúde, o conhecimento e o processo saúde-doença serão comparados antes e após a intervenção nas duas comunidades.

***5.6 Métodos estatísticos***

Todos os dados serão computadorizados em um banco de dados usando o programa Epi Info, versão 6.04d. O cálculo de taxas de prevalência, intervalos de confiança, e testes estatísticos (chi quadrado, Wilcoxon-Mann-Whitney etc.) serão realizados com o mesmo programa.

6. Resultados esperados

Espera-se o controle da tunguíase na área de estudo e o desenvolvimento de medidas de intervenção contra a tunguíase aplicáveis também a outras comunidades no nordeste brasileiro.

7. Considerações éticas

Toda pesquisa deve ser conduzida com três princípios éticos básicos: respeito pela pessoa, benefício para o paciente e justiça.

O respeito pela pessoa trata principalmente do conceito da autonomia, e afirma-se aqui que em nenhuma etapa será ferida a capacidade delas de deliberarem sobre suas escolhas pessoais, já que as informações serão mantidas em sigilo e trabalhadas de forma conjunta e aleatória, após coleta.

O benefício para o paciente refere-se à obrigação ética de maximizar benefícios e minimizar danos e prejuízos. Já firmada a relevância da pesquisa para o Estado e a população, observa-se que inexistem danos ou prejuízos a qualquer uma das partes envolvidas.

Há ainda a justiça, que trata da obrigação ética de tratar cada pessoa com o que é moralmente certo e adequado. Nos estudos propostos, desconhece-se onde este preceito estaria quebrado.

**8. Riscos/benefícios**

O risco do estudo proposto pode ser considerado como muito baixo. A retirada da pulga em condições estéreis raramente pode resultar em infecção secundária. O risco ainda está sendo diminuído pelo uso de antibiótico tópico após a retirada das lesões. Todos os pacientes que apresentarem complicações receberão assistência médica apropriada.

Os participantes serão tratadas. A longo prazo, a tunguíase será controlada na comunidade. As famílias das casas que não têm piso de cimento, receberão material para cimentar o piso após o estudo.

Os resultados trarão informações de grande valor para a Saúde Pública no Estado: será descrita a efetividade de medidas de intervenção contra a tunguíase, problema comum em comunidades pobres, e será descrita a epidemiologia da doença de forma longitudinal.

Qualquer acontecimento desvantajoso para o paciente implicará na saída do mesmo do estudo sem nenhuma desvantagem a respeito do futuro acompanhamento clínico.

Os habitantes da comunidade de controle Barra Velha receberão tratamento contra ectoparasitas e verminoses intestinais após o fim do estudo.

A Fundação Mandacaru se responsabiliza por qualquer dano ao paciente causado pela pesquisa.

**9. Duração da pesquisa e cronograma**

A duração da pesquisa será de 15 meses:

|  | **Oct 02** | **Nov 02** | **Dec 02** | **Jan 03** | **Feb 03** | **Mar 03** | **Apr 03** | **May 03** | **Jun 03** | **Jul 03** | **Aug 03** | **Sep 03** | **Oct 03** | **Nov 03** | **Dec 03** |
| --- | --- | --- | --- | --- | --- | --- | --- | --- | --- | --- | --- | --- | --- | --- | --- |
| Atividades preparatórias |  |  |  |  |  |  |  |  |  |  |  |  |  |  |  |
| Intervenção |  |  |  |  |  |  |  |  |  |  |  |  |  |  |  |
| Monitoramento |  |  |  |  |  |  |  |  |  |  |  |  |  |  |  |

**10. Infraestrutura necessária**

A infraestrutura necessária para esse projeto é mínima. A Fundação Mandacaru alugará uma casa para alojamento dos pesquisadores. A Associação de Desenvolvimento de Balbino (PROBAL) fornecerá uma casa durante o período de intervenção que será usado como centro de informações, escritório e almoxarifado.

**11. Orçamento e financiamento da pesquisa**

A pesquisa será financiada pela Fundação de Educação e Saúde Mandacaru, Fortaleza. Nenhum pesquisador será remunerado.

O gasto total da pesquisa é de R$ 6.900:

| **Item** | **Valor** |
| --- | --- |
| Medicamentos, agulhas etc. | R$1.000 |
| Remuneração de auxiliares | R$2.000 |
| Educação em saúde | R$ 800 |
| Reuniões com comunidade | R$ 500 |
| Aluguel | R$600 |
| Transporte | R$500 |
| Comunicação | R$200 |
| Xerox | R$ 800 |
| Miscelânea | R$500 |
| TOTAL | R$6.900 |

**12. Publicação dos resultados**

Os resultados da pesquisa - favoráveis ou não favoráveis - serão de conhecimento público, sendo apresentados em congressos nacionais e internacionais, além de publicados em jornais indexados nacionais e internacionais.

Como a pesquisa tem a finalidade de uma dissertação do estudante Stefan Schwalfenberg para obtenção do título "Dr. Med.", os resultados também serão publicados em forma de tese.

O material coletado será usado somente para uso específico nesse projeto.

**13. Grupo de pesquisa**

- Dr. Jorg Heukelbach, médico, Fundação de Educação e Saúde Mandacaru, Fortaleza, Ceará: pesquisador responsável
- Stefan Schwalfenberg, estudante de Medicina, Faculdade de Medicina, Universidade Livre de Berlim, Alemanha: pesquisador
- Lars Witt, estudante de Medicina, Faculdade de Medicina, Universidade Livre de Berlim, Alemanha: pesquisador
- Prof. Hermann Feldmeier, Centro de Saúde Internancional, Universidade Livre de Berlim, Alemanha: consultor e orientador
- Dr. Rômulo César Sabóia Moura, médico, Fundação de Educação e Saúde Mandacaru e Posto de Saúde da Família Caponga/Cascavel: consultor

**14. Referências**

1. Hesse,P. Die Ausbreitung des Sandflohs in Afrika. *Geogr Z (Hettner)* 522-530 (1899).

2. Henning,G. Zur Geschichte des Sandflohs (*Sarcopsylla penetrans* L.) in Afrika. *Naturwissenschaftliche Wochenschrift* **20**, 310-312 (1904).

3. Hicks,E.P. The early stages of the jigger,  *Tunga penetrans*. *Ann Trop Med Parasitol* **24**, 575-586 (1930).

4. Gordon,R.M. The jigger flea. *Lancet* **2**, 47-49 (1941).

5. Faust,E.C. & Maxwell,T.A. The findings of the larvae of the chigo, *Tunga penetrans*, in scrapings from the human skin. *Arch Dermatol Syphilol* **22**, 94-97 (1930).

6. Basler,E.A., Stephens,J.H. & Tschen,J.A. *Tunga penetrans*. *Cutis* **42**, 47-48 (1988).

7. Ibanez-Bernal,S. & Velasco-Castrejon,O. New records of human tungiasis in Mexico (Siphonaptera:Tungidae). *Journal of Medical Entomology* **33**, 988-989 (1996).

8. Milgraum,S.S. & Headington,J.T. A subungual nodule of recent onset. Tungiasis. *Arch Dermatol* **124**, 429, 432 (1988).

9. Spielman,M.I., Potter,G.K., Taubman,S.M. & Hodge,W.R. Pain, pruritus, and swelling localized to two toes. Tungiasis. *Arch Dermatol* **122**, 330, 333 (1986).

10. Rietschel,W. Beobachtungen zum Sandfloh (*Tunga penetrans*) bei Mensch und Hund in Französisch-Guayana. *Tierärztliche Praxis* **17**, 189-193 (1989).

11. Oliver Llull,M., Pérez Alfonzo,R. & García,L. Epidemiologia de la *Tunga penetrans* en Venezuela. *Dermatologia Venezuela* **35**, 99-101 (1997).

12. Veraldi,S., Camozzi,S. & Scarabelli,G. Tungiasis presenting with sterile pustular lesions on the hand. *Acta Derm Veneorol* **76**, 495 (1996).

13. Taubman,S.M. & Spielman,M. Tungiasis: a case report. *J Am Podiatr Assoc* **69**, 383-384 (1979).

14. Grosshans,E.M. & Pradinaud,R. Dermatologie in Französisch-Guayana. *Hautarzt* **30**, 443-445 (1979).

15. Zalar,G.L. & Walther,R.R. Infestation by *Tunga penetrans*. *Arch Dermatol* **116**, 80-81 (1980).

16. Mazzini,M.A., Fridmanis,M.I., Obarrio,H.J.A. & Carbajal,G.L. Tungiasis. *Archivos Argentinos de Dermatología* **38**, 403-408 (1988).

17. Chadee,D.D., Furlonge,E., Naraynsingh,C. & Le Maitre,A. Distribution and prevalence of *Tunga penetrans* in coastal south Trinidad, West Indies. *Transactions of the Royal Society of Tropical Medicine and Hygiene* **85**, 549 (1991).

18. Soria,M.F. & Capri,J.J. Tetanos y "piques". *La Prensa Medica Argentina* **40**, 4-11 (1953).

19. Bell,A., Neely,C.L. & Peeples,J. Tungiasis in Tennessee. *South Med J* **72**, 141-143 (1979).

20. Cardoso,A.E.C. Tunguíase. *An Bras Dermatol* **65**, 29S-33S (1990).

21. Matias,R.S. Epidemia de tungíase no Rio Grande do Sul. *Rev Soc Bras Med Trop* **22**, 137-142 (1989).

22. Lowry,M.A., Ownbey,J.L. & McEvoy,P.L. A case of tungiasis. *Mil Med* **161**, 128-129 (1996).

23. Douglas-Jones,A.G., Llewelyn,M.B. & Mills,C.M. Cutaneous infection with *Tunga penetrans*. *British Journal of Dermatology* **133**, 125-127 (1995).

24. Hoeppli,R. Early references to the occurrence of *Tunga penetrans* in Tropical Africa. *Acta Trop* **20**, 143-152 (1963).

25. Sanusi,I.D., Brown,E.B., Shepard,T.G. & Grafton,W.D. Tungiasis: report of one case and review of the 14 reported cases in the United States. *J Am Acad Dermatol* **20**, 941-944 (1989).

26. Fuga,G.C., Dal Fabbro,G., Provini,M. & Ribuffo,A. Osservazioni su tre casi di Tungiasi. *Minerva Medica* **68**, 4115-4120 (1977).

27. Baurle,G. & Stroothenke,M. Tungiasis - eine Urlaubsdermatose. *Hautarzt* **32**, 372-373 (1981).

28. Spradbery,J.P., Bromley,J., Dixon,R. & Tetlow,L. Tungiasis in Australia: an exotic disease threat. *Med J Aust* **161**, 173 (1994).

29. Pfister,R. Nehmen Sandfloh-Infektionen zu? *Fortschritte der Medizin* **95**, 1373-1375 (1977).

30. Arene,F.O. The prevalence of sand flea (*Tunga penetrans*) among primary and post-primary school pupils in Choba area of the Niger Delta. *Public Health*  **98**, 282-283 (1984).

31. Peschlow,I., Schlenzka,K., Merk,G. & Neumann,H.J. Tropendermatosen aktuell. Tungiasis, Ulcus tropicum, Leishmaniase. Beobachtungen aus der Praxis. *Dermatologische Monatsschrift* **169**, 120-124 (1983).

32. Nte,A.R. & Eke,F.U. Jigger infestation in children in a rural area of Rivers State of Nigeria. *West African Journal of Medicine* **14**, 56-58 (1995).

33. Goldsmid,J.M. Tungiasis in Zimbabwe. *Central African Journal of Medicine* **27**, 151-152 (1981).

34. Fimiani,M., Reimann,R., Alessandrini,C. & Miracco,C. Ultrastructural findings in tungiasis. *Int J Dermatol* **29**, 220-222 (1990).

35. Ade-Serrano,M.A. & Ejezie,G.C. Prevalence of tungiasis in Oto-Ijanikin village, Badagry, Lagos State, Nigeria. *Ann Trop Med Parasitol* **75**, 471-472 (1981).

36. Tonge,B.L. Tetanus from chigger flea sores. *J Trop Pediatr* **35**, 94 (1989).

37. Ejezie,G.C. The parasitic diseases of school children in Lagos State, Nigeria. *Acta Trop* **38**, 79-84 (1981).

38. Tan-Lim,K.N. & Pluis,A.H. Tungiasis. *Nederlands Tijdschrift voor Geneeskunde* **116**, 1013-1016 (1972).

39. Pilgrim,R.L. & Brown,G. An instance of tungiasis in New Zealand. *N Z Med J* **106**, 180 (1993).

40. Obengui. La tungose et le tétanos au C.H.U. de Brazzaville. *Dakar Med* **34**, 44-48 (1989).

41. Jeffreys,M.D.W. *Pulex penetrans*: the jigger's arrival and spread in Africa. *South African Journal of Science* **48**, 249-255 (1952).

42. Blanchard,R.A.E. Présence de la chique (*Sarcopsylla penetrans*) à Madagascar. *Archives of Parasitology* **2**, 627-630 (1899).

43. Verhulst,A. *Tunga penetrans* (*Sarcopsylla penetrans*) as a cause of agalactia in sows in the Republic of Zaire. *Veterinary Records* **98**, 384 (1976).

44. Cooper,J.E. An outbreak of *Tunga penetrans* in a pig herd. *Veterinary Records* **80**, 365-366 (1967).

45. Ruthe,H. Fussleiden der Elefanten. *Wissenschaftliche Zeitschrift der Humboldt-Universität zu Berlin, mathematisch-naturwissenschaftliche Reihe* **10**, 474-514 (1961).

46. Heukelbach,J., Wilcke,T., Eisele,M. & Feldmeier,H. Ectopic localizations of tungiasis. *American Journal of Tropical Medicine and Hygiene* **in press**, (2002).

47. Geigy,R. & Herbig,A. Die Hypertrophie der Organe beim Weibchen von *Tunga penetrans*. *Acta Trop* **6**, 246-262 (1949).

48. Feldmeier,H., Heukelbach,J., Eisele,M. & Carvalho C.B.M. Bacterial superinfection in human tungiasis. *Trop Med Int Health* **7**, 559-564 (2002).

49. Chadee,D.D. Tungiasis among five communities in south-western Trinidad, West Indies. *Ann Trop Med Parasitol* **92**, 107-113 (1998).

50. Reiss,F. Tungiasis in New York City. *Arch Dermatol* **93**, 404-407 (1966).

51. Melo,C.R. & Melo,I.S. Linfedema elefantóide verrucoso associado a infestação maciça por *Tunga penetrans*. *An Bras Dermatol* **64**, 35-37 (1989).

52. Mashek,H., Licznerski,B. & Pincus,S. Tungiasis in New York. *Int J Dermatol* **36**, 276-278 (1997).

53. Ade-Serrano,M.A. & Ejezie,G.C. Prevalence of tungiasis in Oto-Ijanikin village, Badagry, Lagos State, Nigeria. *Ann. Trop Med Parasitol.* **75**, 471-472 (1981).

54. Wilcke,T., Heukelbach,J., Cesar Saboia,M.R., Regina,S.K.-P. & Feldmeier,H. High prevalence of tungiasis in a poor neighbourhood in Fortaleza, Northeast Brazil. *Acta Trop* **83**, 255 (2002).

55. Saraceno,E.F. *et al.* Tungiasis: tratamiento de un caso con ivermectina. *Archivos Argentinos de Dermatología* **49**, 91-95 (1999).

56. Youssef,Y.M., Sadaka,H.A.H., Eissa,M.M. & El-Ariny,A.F. Topical application of ivermectin for human ectoparasites. *American Journal of Tropical Medicine and Hygiene* **53**, 652-653 (1995).

57. Ottesen,E.A. & Campbell,W.C. Ivermectin in human medicine. *Journal of Antimicrobial Chemotherapy* **34**, 195-203 (1994).

58. Dunne,C.L., Malone,C.J. & Whitworth,J.A.G. A field study of the effects of ivermectin on ectoparasites of man. *Transactions of the Royal Society of Tropical Medicine and Hygiene* **85**, 550-551 (1991).

59. Caumes,E. *et al.* Efficacy of ivermectin in the therapy of cutaneous larva migrans. *Arch Dermatol* **128**, 994-995 (1992).

60. Cardoso,A. Generalized tungiasis treated with thiabendazole. *Arch Dermatol* **117**, 127 (1981).

1. Franco da Silva,L.A. *et al.* Alguns aspectos epidemiológicos e profiláticos da tungíase em cães de Jataí, GO. *Rev Patol Trop* **30**, 69-73 (2001).

**Anexo 1: termo de consentimento pós-informação**

# TERMO DE CONSENTIMENTO PÓS-INFORMAÇÃO

## I. DADOS SOBRE A PESQUISA CIENTÍFICA

1. TÍTULO DO PROTOCOLO DE PESQUISA:

Tunguíase (*bicho de pé*): controle em uma comunidade severamente afetada no Estado do Ceará

2. PESQUISADORES:

1. Jörg Heukelbach

Profissão: médico

Inscrição no Conselho Regional de Medicina: 7495

2. Stefan Schwalfenberg

Profissão: estudante de Medicina

3. Lars Witt

Profissão: estudante de Medicina

3. AVALIAÇÃO DO RISCO DA PESQUISA:

( ) SEM RISCO (x) RISCO MÍNIMO ( ) RISCO BAIXO ( ) RISCO MÉDIO ( ) RISCO MAIOR (probabilidade de que o indivíduo sofra algum dano como consequência imediata ou tardia do estudo)

4. DURAÇÃO DA PESQUISA: 13 meses

II. REGISTRO DAS EXPLICAÇÕES DO PESQUISADOR AO PACIENTE OU SEU REPRESENTANTE LEGAL SOBRE A PESQUISA, CONSIGNANDO:

1. JUSTIFICATIVA E OBJETIVOS DA PESQUISA:

- controlar o bicho de pé em Balbino
- estabelecer métodos custo-efetivos para controlar o bicho de pé

2. PROCEDIMENTOS QUE SERÃO UTILIZADOS E PROPÓSITOS, INCLUINDO A IDENTIFICAÇÃO DOS PROCEDIMENTOS QUE SÃO EXPERIMENTAIS:

- será realizado exame clínico e serão retirados as pulgas penetradas com uma agulha;

3. DESCONFORTOS E RISCOS ESPERADOS:

O exame clínico será realizado da forma menos incômoda possível para o paciente. Para a retirada das pulgas, será usado álcool para desinfecção, anestesia local com spray, agulha estéril e pomada antibiótica. O risco de superinfecção é baixo por causa de medidas estéreis e da aplicação de antibiótico tópico.

4. BENEFÍCIOS QUE PODERÃO SER OBTIDOS:

O bicho de pé será controlado na comunidade. Todos os habitantes serão tratados.

5. PROCEDIMENTOS ALTERNATIVOS QUE POSSAM SER VANTAJOSOS PARA O INDIVÍDUO:

Não existem procedimentos alternativos mais vantajosos a serem utilizados neste estudo.

III. ESCLARECIMENTOS DADOS PELO PESQUISADOR SOBRE GARANTIAS DO SUJEITO DA PESQUISA

1. o participante pode ter acesso a qualquer tempo às informações sobre procedimentos, riscos e benefícios relacionados à pesquisa, inclusive para esclarecer eventuais dúvidas
2. liberdade de retirar seu consentimento a qualquer momento e deixar de participar do estudo, sem que isto traga prejuízo à continuidade da assistência
3. será mantida a confidencialidade, sigilo e privacidade
4. possibilidade de indenização por eventuais danos à saúde decorrentes da pesquisa

IV. INFORMAÇÕES DE NOMES, ENDEREÇOS E TELEFONES DOS RESPONSÁVEIS PELO ACOMPANHAMENTO DA PESQUISA PARA CONTATO EM CASOS DE INTERCORRÊNCIAS CLÍNICAS E REAÇÕES ADVERSAS

Jörg Heukelbach

Fundação Mandacaru

Rua José Vilar de Andrade 257

Edson Queiroz

Fortaleza, CE 60833-830

Tel. (85) 273.3031

Stefan Schwalfenberg, Lars Witt

Rua Xavier de Castro 96

Praia de Iracema

Fortaleza, CE

Tel. (85) 2192348

V. OBSERVAÇÕES COMPLEMENTARES

VI. CONSENTIMENTO PÓS-ESCLARECIDO

Declaro que, após convenientemente esclarecido pelo pesquisador e ter entendido o que me foi explicado, consinto em participar do presente protocolo de pesquisa. Em caso de menor de idade, declaro que o mesmo foi devidamente esclarecido e aceita participar do presente protocolo de pesquisa sendo eu o responsável legal.

Fortaleza, de de .

_______________________________________/_______________________________

Assinatura do sujeito da pesquisa ou responsável legal (e do menor, quando for possível)

___________________________________________

Assinatura do pesquisador
